# Quantitation of in vivo brain glutathione conformers in cingulate cortex among age‐matched control, MCI, and AD patients using MEGA‐PRESS

**DOI:** 10.1002/hbm.24799

**Published:** 2019-10-04

**Authors:** Deepika Shukla, Pravat Kumar Mandal, Manjari Tripathi, Gayatri Vishwakarma, Ritwick Mishra, Kanika Sandal

**Affiliations:** ^1^ Neuroimaging and Neurospectroscopy (NINS) Laboratory National Brain Research Centre Gurgaon India; ^2^ Florey Institute of Neuroscience and Mental Health Melbourne School of Medicine Campus Melbourne Australia; ^3^ Department of Neurology All India Institute of Medical Sciences New Delhi India; ^4^ Department of Biostatistics Indian Spinal Injuries Centre New Delhi India

**Keywords:** Alzheimer's disease, biomarker, cingulate cortex, closed and extended conformers, glutathione, MEGA‐PRESS, mild cognitive impairment, oxidative stress

## Abstract

Oxidative stress (OS) plays an important role in Alzheimer's disease (AD) and glutathione (GSH) mitigates this effect by maintaining redox‐imbalance and free‐radical neutralization. Quantified brain GSH concentration provides distinct information about OS among age‐matched normal control (NC), mild cognitive impairment (MCI) and AD patients. We report alterations of in vivo GSH conformers, along with the choline, creatine, and *N*‐acetylaspartate levels in the cingulate cortex (CC) containing anterior (ACC) and posterior (PCC) regions of 64 (27 NC, 19 MCI, and 18 AD) participants using MEscher–GArwood‐Point‐RESolved spectroscopy sequence. Result indicated, tissue corrected GSH depletion in PCC among MCI (*p* = .001) and AD (*p* = .028) and in ACC among MCI (*p* = .194) and AD (*p* = .025) as compared to NC. Effects of the group, region, and group × region on GSH with age and gender as covariates were analyzed using a generalized linear model with Bonferroni correction for multiple comparisons. A significant effect of group with GSH depletion in AD and MCI was observed as compared to NC. Receiver operator characteristic (ROC) analysis of GSH level in CC differentiated between MCI and NC groups with an accuracy of 82.8% and 73.5% between AD and NC groups. Multivariate ROC analysis for the combined effect of the GSH alteration in both ACC and PCC regions provided improved diagnostic accuracy of 86.6% for NC to MCI conversion and 76.4% for NC to AD conversion. We conclude that only closed GSH conformer depletion in the ACC and PCC regions is critical and constitute a potential biomarker for AD.

## 1 INTRODUCTION

Alzheimer's disease (AD) is an irreversible, progressive neurodegenerative brain disorder that affects millions of people worldwide. The transition from a normal healthy person to one afflicted with AD is routed through an intermediate state called mild cognitive impairment (MCI) (Almkvist et al., 1998). MCI is described as a heterogeneous group possessing a variety of clinical symptoms with a high risk to convert to AD with a conversion rate of 13.7% per year (Huang, Wahlund, Svensson, Winblad, & Julin, 2002). A recent study, from different continents, also indicated high conversion of both amnesic (aMCI) as well as nonamnestic MCI (naMCI) to AD (Chen, Cheng, Lin, Lee, & Chou, 2018). Although the exact cause of AD is not yet known, recent studies have indicated that oxidative stress (OS) plays a profound role in AD pathogenesis (Mandal, Tripathi, & Sugunan, 2012; Markesbery, 1997; Saharan & Mandal, 2014). Studies have supported increased oxidation in the brain of MCI and AD patients, suggesting high lipid peroxidation and decreased antioxidant defense activities (Guidi et al., 2006; Keller et al., 2005; Padurariu et al., 2010; Swomley & Butterfield, 2015; Torres et al., 2011). Researchers have also indicated biometal homeostasis dysregulation as an important factor for neurodegeneration, where OS resulting from a breakdown in metal‐ion homeostasis leads to an abnormal metal protein chelation pattern (Balmus et al., 2017; Pokusa & Kralova Trancikova, 2017). Reduction in the level of predominant brain antioxidant glutathione (GSH) also found to play a critical role in oxidative imbalance (Bains & Shaw, 1997; Ballatori et al., 2009; Dringen, 2000; Pocernich & Butterfield, 2012). GSH is a master antioxidant and detoxifying agent that is produced de novo in the cytoplasm of human cell. A major proportion of GSH in the human brain is used for radical scavenging and balancing reactive oxygen species (ROS). The diverse functions of GSH originate from the sulfhydryl group in cysteine (Cys), enabling GSH to chelate metals and participate in redox cycling (Jozefczak, Remans, Vangronsveld, & Cuypers, 2012).

The preclinical studies with in vitro and in vivo AD models (Ghosh, LeVault, Barnett, & Brewer, 2012; Labak et al., 2010; Resende et al., 2008; Zhang, Rodriguez, Spaulding, Aw, & Feng, 2012) and an autopsy study conducted on healthy subjects reported that GSH level decreases in the hippocampus (HP) and frontal cortex (FC) with age (Venkateshappa, Harish, Mahadevan, Srinivas Bharath, & Shankar, 2012). Detailed autopsy study involving AD, Parkinson's disease (PD), and dementia with Lewy bodies (DLB) have also presented that the GSH level in the cingulate cortex (CC) region is specifically depleted in AD patients by 49% as compared to age‐matched controls (Gu et al., 1998). These findings emphasize that brain GSH level depletes significantly and specifically in the brain anatomical regions affected in AD pathology. Besides, literature also provides supportive evidence on increased cerebrospinal fluid (CSF) in the brain (Nestor et al., 2008; Silverberg, Mayo, Saul, Fellmann, & McGuire, 2006). The GSH content in the brain CSF in young male (*N* = 26, age 24.6 ± 5.7 years) was observed in negligible amount (0.10–0.20 μM) (Samuelsson, Vainikka, & Ollinger, 2011). However, the GSH content in brain CSF was found to be 0.24 ± 0.07 μM in controls (*N* = 21, M/F 9/12, age 65 ± 10 years) and 0.24 ± 0.10 μM in AD (*N* = 17, M/F 6/11, age 65 ± 8 years) (Konings et al., 1999). Therefore, from these studies, it can be inferred that the GSH amount in brain CSF does not alter in AD pathology. It is interesting to note that the GSH depletion observed in autopsy studies in AD patients was related to the GSH change solely due to brain tissue and not as an atrophic effect of increased CSF. Different imaging studies have shown the concentration of GSH in the human brain is approximately 1–2 mM and this sufficient amount of GSH can also be measured using noninvasive imaging studies (Mandal et al., 2012; Choi, Lee, Denney, & Lynch, 2011; Matsuzawa et al., 2008; Terpstra, Henry, & Gruetter, 2003).

In vivo GSH quantitation using conventional magnetic resonance spectroscopy (MRS) pulse sequence (such as Point‐RESolved spectroscopy [PRESS]) in the brain is challenging and ambiguous due to spectral proximity of CH_2_ peak of Cys (from GSH) with high amplitude creatine (Cr) peak (Duffy et al., 2014; Govindaraju, Young, & Maudsley, 2000; Mandal, 2007; Suri et al., 2017). The advanced spectral editing MEscher–GArwood‐PRESS (MEGA‐PRESS) (Terpstra et al., 2003) pulse sequence has facilitated immensely for selective in vivo GSH detection (Mandal et al., 2012; Terpstra et al., 2003; Trabesinger, Weber, Duc, & Boesiger, 1999) and subsequent quantitation by advanced techniques (Chiang et al., 2017; Mandal, Saharan, Tripathi, & Murari, 2015). Moreover, a recent multicenter study on healthy young controls from different continents has also shown that in vivo GSH exists in two different forms, that is, closed (GSH_cl_) and extended (GSH_ex_) (Mandal et al., 2017), and both can be detected with specific experimental settings using MEGA‐PRESS sequence (Shukla et al., 2018; Shukla, Tripathi, & Mandal, 2018). The brain GSH Cys‐β‐CH_2_ peak at ∼2.80 ppm (identified as closed GSH form) detected using MEGA‐PRESS sequence was quantified for HP and FC regions in AD and MCI patients. It was shown that the amount of GSH varies over different brain anatomical regions in healthy control (HC) subjects and the estimated mean GSH concentration was found to be 1.02 ± 0.17 mM in HP and 1.12 ± 0.18 mM in FC (Mandal et al., 2015). The significant reduction of this closed GSH form in the left hippocampus (LH) of MCI and AD patients, compared to healthy old controls, was suggested as an early diagnostic biomarker for AD (Mandal et al., 2015).

AD pathology is typically characterized by atrophy and hypometabolism within specific brain regions including both anterior cingulate cortex (ACC) and posterior cingulate cortex (PCC) (Bailly et al., 2015). The postmortem (five SuperAgers, five elderly, and five MCI) investigation on the cingulate region also suggested strong correlation (*p* < .05) of neuronal density with the memory capacity in advanced old age participants with better episodic memory function (SuperAgers) than the average elderly and MCI (Gefen et al., 2015). Another autopsy study of definite AD patients (*N* = 31) has also reported a significant positive correlation in apathy and neurofibrillary tangles in ACC brain region (Tekin et al., 2001). Interestingly, within CC, the PCC region as compared with the ACC has been shown to exhibit increased susceptibility to atrophy in familial AD (Jones et al., 2006). Furthermore, some volumetric studies reported AD‐related atrophy in both the ACC and PCC regions of MCI and AD compared with healthy aged subjects (Baron et al., 2001; Bozzali et al., 2006; Frisoni et al., 2002), while other studies reported volumetric loss limited to the PCC region only (Bailly et al., 2015; Callen, Black, Gao, Caldwell, & Szalai, 2001; Shiino et al., 2006). The cingulate area, especially PCC, is one of the earliest regions to exhibit hypometabolism (Mosconi, 2005). Studies on differential susceptibility using PET reported hypometabolism in PCC, but not in the ACC region of early AD (Minoshima et al., 1997; Yokoi et al., 2018) and also in MCI patients (Tomadesso et al., 2015). However, few studies have reported a significant hypometabolism in both the ACC and PCC regions in AD (Bailly et al., 2015; Chetelat et al., 2008; Hauser et al., 2013) as well as in apolipoprotein E (APOE) carriers, when compared to noncarriers (Langbaum et al., 2010). To provide further insight to the biochemical correlates of PCC region activity, a functional magnetic resonance imaging and spectroscopy (fMRI‐MRS) study on healthy participants showed a positive association between the blood oxygen level‐dependent contrast and the total *N*‐acetylaspartate (NAA). However, these findings were neither concurrent with glutamate–glutamine (Glx) and gamma‐aminobutyric acid plus macromolecule (GABA+) concentrations in PCC, nor with occipital metabolite concentrations (Costigan et al., 2018).

Findings from different studies assessing volumetric and metabolic changes in both ACC and PCC regions confirmed their involvement in AD pathology. However, the specific neurochemical susceptibility and their association with OS are still unclear. Hence, it necessitates a further investigation of GSH specific alteration in the both ACC and PCC regions. To the best of our knowledge, there is no study available addressing the GSH alterations in both the ACC and PCC regions of normal control (NC), MCI, and AD using MEGA‐PRESS pulse sequence. Our study probes for the role of GSH conformer in the CC as a potential biomarker for distinguishing MCI and AD from NC. Furthermore, the data processing scheme in this study also incorporates measurement of GSH with a concurrent assessment of other metabolites that is, choline (Cho), creatine (Cr), and NAA from the same MEGA‐PRESS outcome data. Moreover, the quantified absolute GSH levels, as well as tissue corrected GSH levels using partial volume correction (PVC), is also investigated among the three groups. This novel scheme of multiple metabolite detection and quantitation from single noninvasive MRS data acquisition broadens the use of MEGA‐PRESS pulse sequence in clinical setting.

## 2 METHODS

### 2.1 Participant recruitment

A total of 64 (27 NC; 19 MCI and 18 AD) participants including both male (M) and female (F) were recruited for this cross‐sectional study (Table 1). The NC participants were recruited from the HelpAge India and patients diagnosed with MCI and AD were recruited by the neurologist (MT) from Out Patient Department (OPD), Department of Neurology, All India Institute of Medical Sciences (AIIMS), New Delhi. The MCI patients were diagnosed as per the revised Petersen criteria (Gauthier et al., 2006; Petersen et al., 1999) and the memory registration, visuospatial, object recognition, attention, recall, procedural memory, and language domains were tested for this study. Likewise, the AD patients were diagnosed as per the National Institute of Neurological and Communicative Disorders and Stroke and the AD and Related Disorders Association revised criteria for the clinical diagnosis of probable AD (McKhann et al., 1984; McKhann et al., 2011). The eligibility criteria for all participants were age 55 years and above, and absence of any other neurological or psychiatric condition known to impact brain function and associated outcome. The participants with any known contraindication toward MRI (metallic implants or claustrophobic) were excluded from this study. The study was approved by the Institutional Human Ethics Committee at the National Brain Research Centre (NBRC), Gurgaon. The purpose of the study was well explained to all the participants and/or to the accompanying relatives before written informed consent was obtained.

**Table 1:** Demographic characteristics and metabolic measurements

Variable	NC	MCI	AD	Test statistics	*p*‐value
Demographic characteristics					
No. participants (*N*)	27	19	18	—	—
Age^a^ (years)	68.44 ± 7.07	70.84 ± 7.74	70.74 ± 8.33	*F*(2) = 0.749	.477
Gender (M/F)	16/11	16/3	10/8	*χ* ^2^ = 4.203	.122
Metabolic measurements^a^^,^ b ^,^ c					
ACC					
PVC GSH_cl_ concentration	1.44 ± 0.27* (25)	1.27 ± 0.30* (18)	1.17 ± 0.41* (17)	*F*(2) = 3.878	.026
GSH_cl_ concentration	1.9502 ± 0.4223** (26)	1.6224 ± 0.3865** (18)	1.5624 ± 0.3407** (16)	*F*(2) = 6.187	.004
GSH_cl_ peak area	0.0029 ± 0.0008** (26)	0.0022 ± 0.0007** (18)	0.0021 ± 0.0021** (16)	*F*(2) = 6.187	.004
NAA peak area	0.0016 ± 0.0004*** (27)	0.0012 ± 0.0001*** (17)	0.0013 ± 0.0002*** (16)	*F*(2) = 11.345	<.001
Cr peak area	0.0014 ± 0.0002*** (27)	0.0011 ± 0.0001*** (17)	0.0011 ± 0.0002*** (16)	*F*(2) = 11.228	<.001
Cho peak area	0.0013 ± 0.0003*** (27)	0.0009 ± 0.0002*** (17)	0.0011 ± 0.0002*** (16)	*F*(2) = 12.138	<.001
PCC					
PVC GSH_cl_ concentration	1.60 ± 0.25** (24)	1.28 ± 0.25** (16)	1.37 ± 0.29** (14)	*F*(2) = 8.191	.001
GSH_cl_ concentration	2.3674 ± 0.4049*** (25)	1.7708 ± 0.3444*** (16)	1.7794 ± 0.4361*** (16)	*F*(2) = 15.515	.001
GSH_cl_ peak area	0.0037 ± 0.0007*** (25)	0.0025 ± 0.0006*** (16)	0.0025 ± 0.0009*** (16)	*F*(2) = 15.515	<.001
NAA peak area	0.0019 ± 0.0003*** (24)	0.0015 ± 0.0001*** (15)	0.0014 ± 0.0002*** (14)	*F*(2) = 21.275	<.001
Cr peak area	0.0014 ± 0.0002*** (24)	0.0011 ± 0.0001*** (15)	0.0012 ± 0.0001*** (14)	*F*(2) = 15.653	<.001
Cho peak area	0.0008 ± 0.0002*** (24)	0.0007 ± 0.0001*** (15)	0.0007 ± 0.0001*** (14)	*F*(2) = 10.146	<.001
CINGULATE					
PVC GSH_cl_ concentration	3.06 ± 0.43** (23)	2.46 ± 0.42** (15)	2.55 ± 0.60** (14)	*F*(2) = 8.908	.001
GSH_cl_ concentration	4.3221 ± 0.7052*** (24)	3.3019 ± 0.5658*** (15)	3.3848 ± 0.6609*** (14)	*F*(2) = 14.661	<.001
GSH_cl_ peak area	0.0066 ± 0.0013*** (24)	0.0046 ± 0.0011*** (15)	0.0046 ± 0.0015*** (14)	*F*(2) = 14.083	<.001
NAA peak area	0.0036 ± 0.0006*** (24)	0.0027 ± 0.0002*** (15)	0.0028 ± 0.0004*** (14)	*F*(2) = 17.039	<.001
Cr peak area	0.0028 ± 0.0004*** (24)	0.0022 ± 0.0002*** (15)	0.0024 ± 0.0002*** (14)	*F*(2) = 14.392	<.001
Cho peak area	0.0022 ± 0.0004*** (23)	0.0016 ± 0.0002*** (15)	0.0018 ± 0.0002*** (14)	*F*(2) = 13.206	<.001

Abbreviations: ACC, anterior cingulate cortex; AD, Alzheimer's disease; AUC, area under curve; GSH, glutathione; MCI, mild cognitive impairment; NC, normal control; PCC, posterior cingulate cortex; PVC, partial volume correction; ROC, receiver operator characteristic.

a All variables are represented as mean ± *SD* (*N*), with peak area value in arbitrary unit (a.u.) and GSH concentration in mM.

b All variables were tested for homoscedasticity using Levene's test.

c All variables were tested for normality distribution using Shapiro–Wilk test.

All significant values were set at *p* < .05 (*p < .05, **p < .01, ***p < .001).

### 2.2 MRI and MRS data acquisition

**Figure 1:**
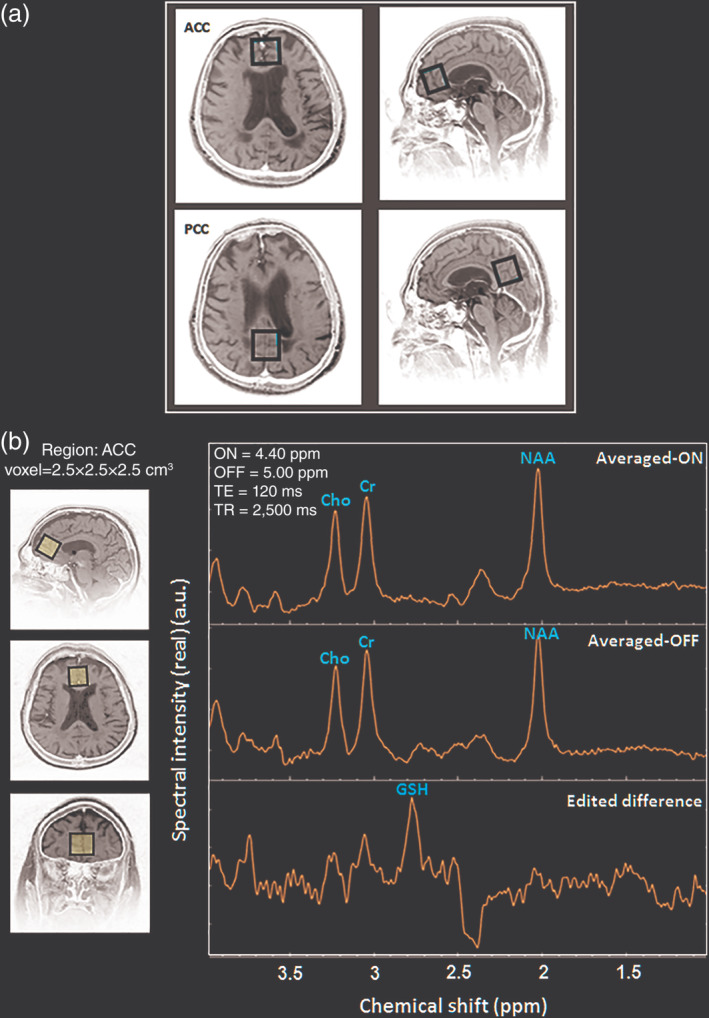
(a) Demonstration of MRS voxel placement of size 2.5 × 2.5 × 2.5 cm^3^ on the ACC and PCC regions. (b) In vivo brain GSH detection using MEGA‐PRESS (ON = 4.40 ppm; OFF = 5.00 ppm, TE = 120 ms, TR = 2,500 ms, NSA = 16) in the ACC region (Averaged‐ON, averaged‐OFF, and edited‐difference spectra). Abbreviations: ACC, anterior cingulate cortex; GSH, glutathione; MEGA‐PRESS, MEscher‐GArwood‐Point‐RESolved spectroscopy; MRS, magnetic resonance spectroscopy; NSA, number of spectral acquisitions; PCC, posterior cingulate cortex

All proton (^1^H) MRI/MRS data on human and phantom were acquired at NBRC with a 3 T MR scanner (Achieva, Philips, Netherlands) equipped with a dual tuned (^1^H/^31^P) transmit/receive volume head coil (Rapid GmbH, Germany). Scout images were obtained in axial, sagittal, as well as coronal planes. In order to aid the anatomical localization, T_2_‐weighted MRI images with turbo spin echo were acquired in all the three planes with the following acquisition parameters: repetition time (TR) = 3,000 ms; echo time (TE) = 80 ms; flip angle = 90°; and turbo factor = 15. The MRI images were used as a visual reference for voxel placement on the ACC and the PCC regions (Figure 1a). GSH phantom as well as in vivo MRI and MRS experiments in this study were carried out by one of us (P.K.M.) in order to have consistency in voxel positioning and data quality. In vivo voxels were placed based on the anatomical referencing taken as its adjacency to the corpus callosum, and also good shimming for data quality control was ensured during data acquisition.

Both in vivo and phantom GSH data were acquired using the MEGA‐PRESS pulse sequence. In order to selectively refocus the evolution of J‐coupled GSH spins (Hβ–Cys) at 2.80 ppm, the editing pulse was applied to GSH spins (Hα–Cys) at 4.40 ppm (referred to as “ON”) (Delalande et al., 2010; Shukla, Mandal, et al., 2018; Shukla, Tripathi, & Mandal, 2018). In another set of data acquisition using MEGA‐PRESS, the inversion pulse was applied at 5.00 ppm (referred to as “OFF”) so as J‐coupling evolves freely throughout the TE without affecting the GSH peak. All MRS data having 2,048 sample points were acquired with TE = 120 ms and TR = 2,500 ms. The optimal value of TE (120 ms) is considered for the GSH quantitation as it provides improved signal to noise ratio (SNR) (Chan et al., 2017; Mandal et al., 2015). The 20 interleaved spectral dynamics (each as an average of 16 number of spectral acquisitions) were acquired with ON pulse set at 4.40 ppm (MEGA‐ON), followed by OFF pulse set at 5.00 ppm (MEGA‐OFF). In MEGA‐PRESS sequence, the excitation 90° pulse and the refocusing 180° pulse bandwidths were set at 2.4 and 1.2 kHz, respectively. Detection of other brain metabolites, that is, NAA, Cr, and Cho were presented from MEGA‐OFF spectra which has TE = 120 ms same as of edited‐difference MEGA‐PRESS spectra for GSH quantitation. The MRS scan time for each MEGA‐PRESS scan was 14 min and 02 sec. In studies involving AD patients, maintenance of total scan time is extremely crucial considering the comfortability of AD patients and thus, the data acquisition for additional neuronal markers such as NAA, Cr, and Cho using traditional PRESS sequence is very challenging. The present methodology of quantifying both MEGA‐OFF as well as edited‐difference spectra presents the capability of MEGA‐PRESS sequence for quantifying multiple metabolites from single MRS data acquisition. Water suppression in each MRS data was accomplished with the Chemical Shift Selective Suppression pulse sequence (Haase, Frahm, Hanicke, & Matthaei, 1985). Shimming was achieved using pencil beam–volume resulting in water linewidth of ≤15 Hz for both ACC and PCC regions. The region of interest, with a voxel size of 2.5 × 2.5 × 2.5 cm^3^ (volume 15.6 cc) was selected in the center of the phantom for in vitro and over the ACC and the PCC regions for in vivo data collection (Figure 1a). in vivo single voxel MRS planning was carried out by positioning the voxel over the cingulate gyrus, adjacent to the corpus callosum in frontal and parietal lobes. This ensures consistency by concurrently checking the axial, coronal, and sagittal planes.

### 2.3 GSH MEGA‐PRESS spectroscopy data processing

MEGA‐PRESS data were processed using our in‐house developed MATLAB (The MathWorks, Inc. Natick, MA) based, MRS signal processing and analysis toolbox, KALPANA (Mandal et al., 2015; Mikkelsen et al., 2019). All data processing and analysis were performed by one of us (D.S.) keeping the clinical status of participants blinded. The MRS processing pipeline included a quantitative assessment of signal quality as well as visual inspection of each subspectra, where the data that had poor signal quality due to lipid or head movement artifacts were rejected. The data quality of raw MEGA‐PRESS data in terms of SNR of each individual, ON as well as OFF spectra was calculated in frequency domain within range of 0.5–6.0 ppm using third level wavelet decomposition (Mallat, 1989). In this, the signal within 2.85–3.15 ppm range refers to the “Cr peak” whereas, the noise is considered as the real value of 500 points of lower end of frequency domain of Free Induction Decay (FID) signal which had no known metabolite. Starting assignment of each singlet peak in the ^1^H spectra was set with an appropriate frequency shift and defined Gaussian line‐shape. Frequency referencing was performed with respect to water peak aligned at 4.67 ppm. Furthermore, the spectra were preprocessed by autophase correction. The first time‐domain data point of FID of each spectral dynamic was used for zero‐order phase estimation and same applied over all the MEGA‐ON and MEGA‐OFF dynamics of MEGA‐PRESS data in this implementation. The interleaved sum of each of the phase‐corrected dynamics (total 10) gives the average of all the MEGA‐ON (averaged‐ON) and the MEGA‐OFF (averaged‐OFF) spectra separately. The difference between the averaged‐ON and averaged‐OFF, that is, edited difference, was used for the GSH peak area estimation and quantitation. Spectral apodization was performed over the time domain data using mixed Gaussian and exponential window functions, and subsequently, Fourier transformation was applied on the apodized time‐domain data. In addition, manual zero‐order phase correction was applied on the edited difference and the averaged‐OFF spectrums to ensure the correct GSH and other metabolite peak areas estimation, respectively. Selective peak suppression of the residual water (−70 Hz, 70 Hz) and lipid (−480 Hz, −520 Hz) was performed using Hankel–Lanczos singular value decomposition filtering method (Pijnappel, Boogaart, Beer, & Ormondt, 1992). Singular spectrum analysis (SSA) was applied for spectral baseline estimation and further, spectral fitting was performed using time‐domain nonlinear least square cost function optimization (Golyandina & Shlemov, 2015).

**Figure 2:**
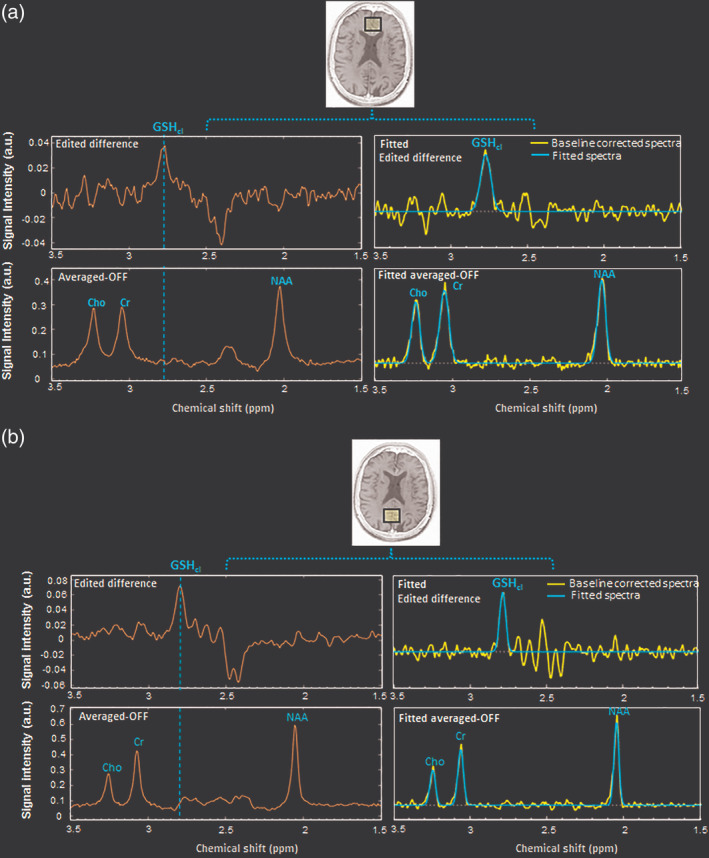
Detection and quantitation of in vivo GSH‐closed form (GSH_cl_) using edited‐difference and other ^1^H‐MRS metabolite peaks of Cho, Cr, and NAA using the averaged‐OFF spectra of MEGA‐PRESS data with fitted spectral peaks in the ROI as (a) ACC and (b) PCC regions of an NC participant. Abbreviations: ACC, anterior cingulate cortex; GSH, glutathione; MEGA‐PRESS, MEscher‐GArwood‐Point‐RESolved spectroscopy; MRS, magnetic resonance spectroscopy; NAA, *N*‐acetylaspartate; NC, normal control; PCC, posterior cingulate cortex; ROI, region of interest

**Figure 3:**
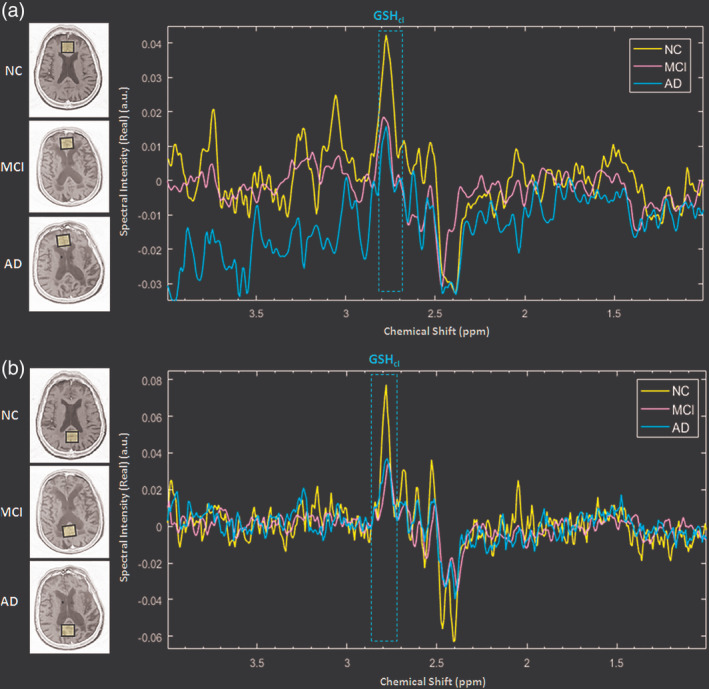
Illustration of comparative amount of GSH‐closed form (GSH_cl_) peak (dotted box) obtained from the (a) ACC and (b) PCC regions in the age‐matched control (NC), MCI, and AD participant. Abbreviations: ACC, anterior cingulate cortex; AD, Alzheimer's disease; GSH, glutathione; MCI, mild cognitive impairment; NC, normal control; PCC, posterior cingulate cortex

In vivo GSH from both ACC and PCC, ROIs were detected with edited‐difference spectra of MEGA‐PRESS sequence by application of refocusing 180° pulse at 4.40 ppm, and the obtained GSH_cl_ peak at ∼2.80 ppm was referred as the Cys–β‐CH_2_ resonance of GSH (Mandal, Shukla, Govind, Boulard, & Ersland, 2017;Shukla, Mandal, et al., 2018 ; Shukla, Tripathi, & Mandal, 2018) (Figure 1(b)). Additionally, GSH_ex_ peak at ∼2.95 ppm was observed by phase correcting the same edited‐difference spectra (Shukla, Mandal, et al., 2018; Shukla, Tripathi, & Mandal, 2018) (Figure S1). The GSH_cl_ at ∼2.80 ppm in the edited‐difference spectrum and the Cho, Cr, and NAA peaks in the averaged‐OFF spectrum were fitted with prior knowledge; including the frequency shift and the Gaussian line‐shape. The spectral peak fitting was combined with an iterative baseline estimation scheme to achieve minimum residue between the baseline‐corrected spectra and the peak fitted model (Mandal et al., 2012; Young, Soher, & Maudsley, 1998). This approach has previously been shown to yield a better quantitation result as compared to a single pass optimization method that models baseline and metabolite signal together (Soher, Young, & Maudsley, 2001). Metabolite peak area estimation for GSH_cl_ (using edited difference), and Cho, Cr, and NAA (using averaged‐OFF) in ACC and PCC regions of a single NC participant is presented in Figure 2. Relative peak areas of Cho, Cr, and NAA obtained from PRESS (TE = 41 ms) and averaged‐OFF spectra of MEGA‐PRESS sequence (TE = 120 ms and TE = 130 ms) were also compared and found to retain a similar trend over the change in TE values (Figure S2). Quality of fitted GSH_cl_ peak among, NC, MCI, and AD groups were assessed in the frequency domain in terms of full‐width‐at‐half maximum (FWHM), SNR, and Cramer Rao Lower Bound (CRLB) (Cavassila, Deval, Huegen, van Ormondt, & Graveron‐Demilly, 2001). SNR for fitted edited‐MEGA spectra within range of 1.0–4.0 ppm was obtained taking an average of the fitted model as a signal, whereas the residual error between fitted model and the processed edited spectra was considered as noise. GSH peak quality assessment based on this SNR is also reflected in the CRLB calculation that is more directly linked to the confidence limits. Moreover, in the literature, CRLB has been suggested as a better indicator than SNR for judging the data quality (Kreis, 2004; Matsuda, Asada, & Tokumaru, 2017). All quality measures, as well as associative information on MRS metabolite peaks, are presented in terms of mean ± *SD*. Spectral quality in terms of FWHM (9.61 ± 2.69), SNR (3.48 ± 1.86), and CRLB (10.84 ± 3.80) for GSH_cl_ peak was measured for both the ACC and the PCC regions in the three study groups. Individual data having CRLB value of less than 20% for fitted GSH_cl_ peak was rejected in this study. The GSH_cl_ peak from both ACC (Figure 3a) and PCC (Figure 3b) regions among the NC, MCI, and AD participants shows a sharp decline from NC to MCI transition, while no appreciable change was observed for MCI to AD transition. Fitted spectra for these GSH_cl_ peaks and their calculated area values for both ACC and PCC regions in NC, MCI and, AD participants (same as in Figure 3), are also presented in Figure S3.

### 2.4 Absolute quantitation

The absolute quantitation of in vivo GSH was performed with external calibrated referencing using GSH phantom, where GSH (Sigma Aldrich) solution was kept in a spherical phantom (diameter = 17 cm) containing 1–5 mM/L. The GSH solution in a phosphate buffer at neutral pH 7.2 was used to overcome the loading factors and similar receiver gain was maintained in phantom and in vivo studies. The in vitro GSH signal area was found to be linearly dependent on actual GSH concentration values, with a coefficient of determination *R* ^2^ = 0.994 (Figure S4). The absolute GSH concentration ([Absolute_GSH_conc_]) in mM was estimated by applying *T*_1_ and *T*_2_ relaxation time correction factors on the estimated GSH peak area (Area_GSH_) from edited‐difference spectra. The conversion of signal peak area to concentration was performed using the slope (*m*) and intercept (*k*) of the fitted line equation of phantom calibration curve as given in Figure S4. The external reference for the absolute concentration of in vivo GSH level was obtained from GSH phantom calibration curve using Equation (1):

(1) $$\left[{\text{Absolute}}_{\mathrm{GSH}\_\text{conc}}\right]=\frac{{\text{Area}}_{\mathrm{GSH}}-k}{m}\times \frac{1-\exp\left(-\frac{\mathrm{TR}}{T1_{\text{Phantom}}}\right)}{1-\exp\left(-\frac{\mathrm{TR}}{T1_{\mathrm{GSH}}}\right)}\times \frac{\exp\left(-\frac{\mathrm{TE}}{T2_{\text{Phantom}}}\right)}{\exp\left(-\frac{\mathrm{TE}}{T2_{\mathrm{GSH}}}\right)}$$

The relaxation times (*T*_1_ and *T*_2_) of GSH for both phantom and in vivo were inferred from published literature for 3 T system as follows: *T*_1GSH_ (397) (Choi & Lee, 2013), *T*_1Phantom_ (350 ms) (Sanaei Nezhad, Anton, Parkes, Deakin, & Williams, 2017), *T*_2GSH_ (117 ms) (from Andreas Hock email communication to P.K.M.) (Scheidegger, Hock, Fuchs, & Henning, 2014), and *T*_2Phantom_ (95 ms). Mean values of mentioned *T*_1_ and *T*_2_ were used to calculate the respective correction factors as appropriate. TE = 120 ms and TR = 2,500 ms, while *m* and *k* of linearly fitted calibration curve were taken as 0.00245 and −0.00091, respectively.

### 2.5 PVC for CSF contamination within MRS voxel

In AD pathology, brain CSF increases significantly within the lateral ventricle and nearby regions (Nestor et al., 2008; Silverberg et al., 2006). Existing literature has also supported the negligible amount of GSH within brain CSF, and only gray matter (GM) and white matter (WM) brain tissues contain the antioxidant GSH (Konings et al., 1999). Thus, the tissue degeneration within a voxel, as well as the presence of CSF in ACC and PCC regions, can potentially introduce inaccuracy in the GSH quantitation. In order to eliminate the effect of increased CSF or tissue degeneration within MRS voxels placed at the ACC and PCC regions, a PVC methodology, given in Equation (2) was applied to obtain the tissue or partial volume corrected GSH levels ([PVC_GSH_conc_]). The method used here considers the partial volume effect of only CSF (McLean, Woermann, Barker, & Duncan, 2000; Quadrelli, Mountford, & Ramadan, 2016). In this study, the tissue segmentation on the T_2_‐weighted axial MRI slices was performed using statistical parametric mapping software (SPM 12), and the GSH concentration results were corrected for CSF contamination in the MRS voxel for both ACC and PCC regions. Binary MRS voxel masks were coregistered over segmented tissue maps to give the relative proportion of GM, WM, and CSF within each voxel. The segmentation of the MRI and calculation of PVC correction factors were performed using our in‐house package KALPANA (Mandal et al., 2015; Mikkelsen et al., 2019) and absolute tissue corrected GSH levels within MRS voxels (PVC_GSH_conc_) were calculated using Equation (2).

(2) $$\left[{\mathrm{PVC}}_{\mathrm{GSH}\_\text{conc}}\right]=\left[{\text{Absolute}}_{\mathrm{GSH}\_\text{conc}}\right]\times \left(\frac{1}{1-V_{\mathrm{csf}}}\right)$$

where [Absolute_GSH_conc_] is the relaxation time corrected absolute GSH concentration (in mM) obtained using KALPANA package (Mandal et al., 2015; Mikkelsen et al., 2019), which was calibrated using known GSH concertation levels in the spherical phantom and corrected using respective relaxation times. The volumetric fraction of the CSF (*V*_csf_) contained within the MRS voxel was obtained by applying binary voxel mask coregistration over segmented tissue and CSF planes generated from SPM 12.

### 2.6 Statistical analysis

Differences in demographic characteristic variables (i.e., age and gender) and metabolic measurements (i.e., peak areas and concentrations) among participant groups (NC, MCI, and AD) were assessed using *χ* ^2^ test for categorical data and one‐way analysis of variance (ANOVA [*F*(2), *p*‐value]) or Kruskal–Wallis ANOVA (*H*(2), *p*‐value) for continuous variables as appropriate. Normality and homoscedasticity for all metabolite levels were assessed using the Shapiro–Wilk test and Levene's test (*W*(2), *p*‐value), respectively. The effect of clinical status on brain metabolite levels was assessed by the use of one‐way ANOVA, followed by Tukey test for pairwise post hoc comparisons. The association between GSH level and factors (group, region, and group × region) was assessed using a generalized linear model (GLM) including age and gender as covariates. Adjustment for multiple comparisons was performed using Bonferroni correction. Significance levels for all the statistical analyses were set at *p* < .05. Statistical analyses were performed using IBM SPSS Statistics V25.0.

To evaluate, compare, and differentiate among the three groups (NC, MCI, and AD) in accordance with GSH level in specific brain region, receiver operator characteristic (ROC) curve analyses were performed using DeLong criteria (DeLong, DeLong, & Clarke‐Pearson, 1988). Additionally, multivariate ROC curve analysis was performed on the predicted probability values, obtained using binary logistic regression on the GSH_cl_ concentration levels from both ACC and PCC regions together. Optimal cut‐off values for ROC curves were determined using the Youden index criteria. Corresponding area under curves (AUCs), sensitivity, specificity, 95% confidence intervals (CI), and accuracy were estimated and presented with *χ* ^2^ statistics to determine the significance of the model. The effect of clinical status on GSH level was assessed in the individual ACC, PCC, and CINGULATE regions (considering ACC and PCC regions together from the same participant). The GSH_cl_ peak area, absolute relaxation time corrected GSH levels and the tissue corrected GSH concentration levels were considered for all three regions (ACC, PCC, and CINGULATE) as a within‐subject fixed factor, and between‐subject fixed factor for the three groups (NC, MCI, and AD). Age and gender were considered as two covariates to analyze the main effect of cingulate GSH level on the three groups.

## 3 RESULTS

### 3.1 Demographic characteristics of participant groups

The demographic characteristics of the participant groups are presented in Table 1. The mean age of the study participants (*N* = 64) was 69.81 ± 7.61 years with 42 males and 22 females. There was no significant difference in age (*p* = .477), and gender (*p* = .122) among the three groups (Table 1).

### 3.2 Metabolic alterations in the cingulate cortices of NC, MCI, and AD groups

To assess the relationship of GSH and other neuronal markers (Cho, Cr, and NAA) with the progressive disease severity in MCI and AD pathology, the mean ± *SD* of these metabolites peak areas were analyzed for both the ACC and PCC regions (Table 1). Pairwise comparisons between the groups revealed a significant decrease in GSH_cl_ peak area levels of the CC region in both AD (*p* < .001) and MCI (*p* < .001) as compared to NC, whereas no significant difference (*p* = .999) between MCI and AD was observed. The results suggest the involvement of the CC region in early pathogenesis from NC to MCI conversion. Independent GSH_cl_ peak area level using one‐way ANOVA followed by post hoc analysis showed a significant change in the PCC and the ACC region between NC‐MCI (PCC: *p* < .001; ACC: *p* = .023), and NC‐AD (PCC: *p* < .001; ACC: *p* = .008) groups; however, no significant change was observed between MCI‐AD groups (PCC: *p* = .998; ACC: *p* = .897). The post hoc comparison signifies the higher susceptibility of OS over PCC region than the ACC region at an early stage of the disease. Figure S3a,b shows the GSH_cl_ peak areas of NC, MCI and, AD participants from the PCC and the ACC regions, respectively. Apart from this, peak area levels of GSH_ex_ estimated from phase corrected edited MEGA‐PRESS spectra do not reveal any significant variation among the three groups (Figure S1b). The GSH_ex_ conformer peak from the experimental protocol used for MRS in this study (i.e., TE = 120 ms, TR = 2,500 ms, ON = 4.40 ppm, and OFF = 5.0 ppm) was indistinct and needs to be further investigated. The existence of the two conformers using both the TEs (i.e., 120 and 130 ms) and the combination of ON pulses at 4.40 ppm as well as 4.56 ppm in different brain regions have already been established in our recently conducted multicenter in vivo MR studies (Mandal et al., 2017; Shukla, Mandal, et al., 2018; Shukla, Tripathi, & Mandal, 2018). Investigation on the relative alteration of each of the two forms of reduced GSH in healthy aging as well as pathological conditions is critical in disease understanding and is an active area of current research at Neuroimaging and Neurospectroscopy (NINS) laboratory, NBRC, India.

**Figure 4:**
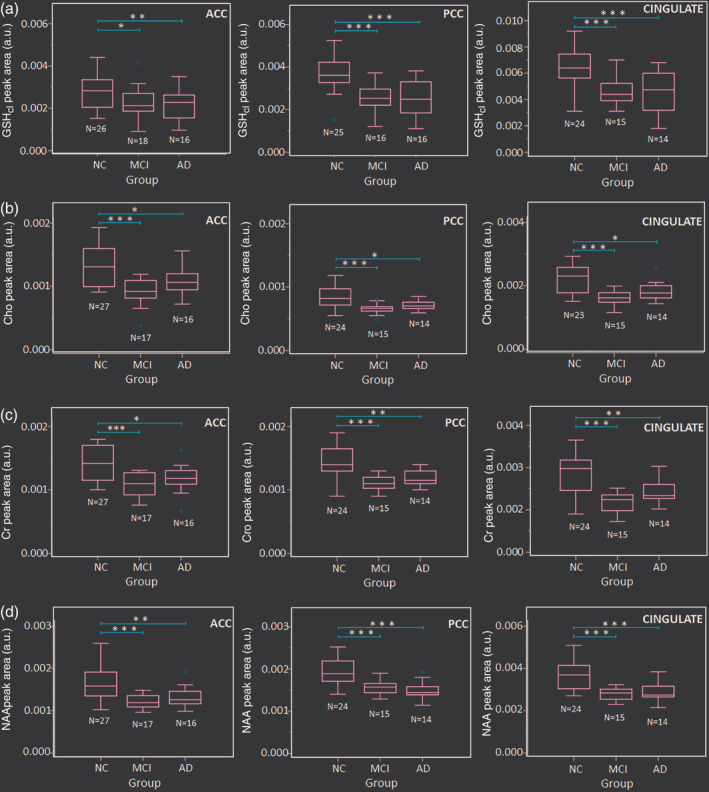
Box plots for quantified area values of (a) GSH‐closed form (GSH_cl_), and (b) Cho, (c) Cr, and (d) NAA as ^1^H‐MRS metabolites obtained from MEGA‐PRESS sequence in the ACC, PCC, and CINGULATE (includes ACC and PCC regions from the same participant) regions from age‐matched control (NC), MCI, and AD participants (*p < .05, **p < .01, ***p < .001). Abbreviations: ACC, anterior cingulate cortex; AD, Alzheimer's disease; GSH, glutathione; MCI, mild cognitive impairment; MEGA‐PRESS, MEscher‐GArwood‐Point‐RESolved spectroscopy; MRS, magnetic resonance spectroscopy; NAA, *N*‐acetylaspartate; NC, normal control; PCC, posterior cingulate cortex

Apart from GSH, other ^1^H‐MRS metabolite peak areas for Cho, Cr, and NAA obtained from averaged‐OFF spectra were also evaluated using ANOVA for between‐group changes. Post hoc analysis result revealed significant decrease in all these three metabolites between NC‐MCI [ACC: (Cho: *p* < .001; Cr: *p* < .001 and NAA: *p* < .001); PCC: (Cho: *p* < .001; Cr: *p* < .001 and NAA: *p* < .001); CINGULATE: (Cho: *p* < .001; Cr: *p* < .001 and NAA: *p* < .001)] and NC‐AD [ACC: (Cho: *p* = .013; Cr: *p* = .010 and NAA: *p* = .003); PCC: (Cho: *p* = .014; Cr: *p* = .001; and NAA: *p* < .001); CINGULATE: (Cho: *p* = .012; Cr: *p* = .006; and NAA: *p* < .001)] groups. However, no significant changes were observed for disease progression from MCI to AD (Figure 4 and Table 1). Alike GSH depletion, with increased OS, the changes in Cho, Cr, and NAA metabolites were not specific to AD pathology and findings from literature remain inconsistent on the changes of these metabolites in normal aging as well as in pathological conditions.

### 3.3 GSH alteration in ACC, PCC, and CINGULATE regions among NC, MCI, and AD groups

**Figure 5:**
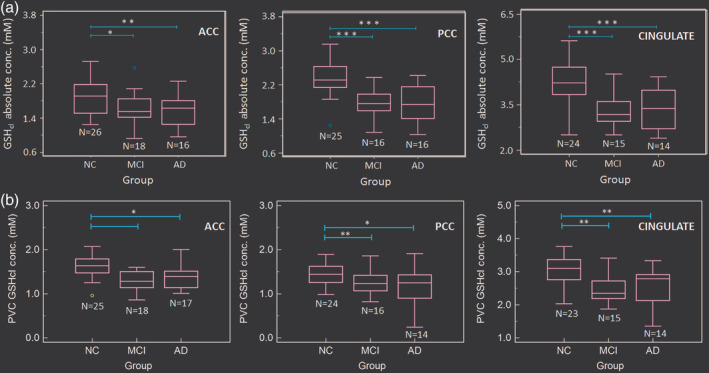
Box‐plots for absolute concentration values of (a) GSH‐closed form (GSH_cl_) and (b) PVC–GSH_cl_ in the ACC, PCC, and CINGULATE (includes ACC and PCC regions from the same participant) regions from age‐matched control (NC), MCI, and AD participants (*p < .05, **p < .01, ***p < .001). Abbreviations: ACC, anterior cingulate cortex; AD, Alzheimer's disease; GSH, glutathione; MCI, mild cognitive impairment; NC, normal control; PCC, posterior cingulate cortex; PVC, partial volume correction

One‐way ANOVA of both *T*_1_ and *T*_2_ corrected absolute GSH_cl_, as well as, partial volume or tissue corrected (PVC–GSH) concentration levels revealed a significant difference among NC, MCI, and AD groups (Table 1). Post hoc analysis of the absolute GSH_cl_ concentration levels in the ACC, PCC, and CC regions were found to be significantly depleted in MCI (PCC: *p* < .001, ACC: *p* = .023, and CINGULATE: *p* < .001) and AD (PCC: *p* < .001, ACC: *p* = .008, and CINGULATE: *p* < .001) groups as compared to NC (Table 1). The results in ACC region showed a gradual depletion of absolute GSH_cl_ concentration with disease progression, whereas in PCC, a significant decline was observed at an early stage of the disease, that is, for NC to MCI transition (Figure 5a). However, no significant difference in the GSH_cl_ level between MCI to AD (PCC: *p* = .998, ACC: *p* = .897, CINGULATE: *p* = .938) was observed. Post hoc analysis of the PVC–GSH concentration levels obtained using Equation (2) showed significant depletion in PCC region for both MCI (*p* = .001) and AD (*p* = .028) groups as compared to NC, while ACC region showed such changes only for AD (*p* = .025) but not for MCI (*p* = .194) as compared to NC group (Figure 5b, Table 1). The cingulate GSH levels as a combination of ACC and PCC together reflect significant changes in both MCI (*p* = .001) and AD (*p* = .008) groups as compared to NC. None of the regions showed significant difference in tissue corrected GSH levels between MCI and AD groups (PCC: *p* = .615, ACC: *p* = .643, and CC: *p* = .854).

### 3.4 Effect of groups, brain regions, and their interaction on the GSH concentration levels

The effects of group (i.e., NC, MCI, and AD), region (i.e., ACC, PCC, and CINGULATE), and group × region interaction on absolute GSH levels (Table 2) as well as tissue corrected GSH levels were assessed with age and gender as covariates (Table 3). There was significant effect on absolute GSH levels due to group (*p* < .001), region (*p* < .001) and their interaction, that is, group × region (*p* = .004), whereas the PVC–GSH levels showed significant effect due to group (*p* < .001) and region (*p* < .001) but not in group × region (*p* = .431). Pairwise comparisons between the groups showed significant decrease in absolute GSH levels in both MCI (MD = 0.684, *p* < .001) and AD (MD = 0.534, *p* < .001) as compared to NC, where, MD is a mean‐difference value (Table 4). Similarly, pairwise comparisons between the regions revealed significant decrease in GSH levels in both ACC (MD = 1.977, *p* < .001) and PCC (MD = 1.706, *p* < .001) region as compared to CINGULATE region (Table 5). Pairwise comparisons of PVC–GSH levels between the groups showed significant decrease in GSH levels in both MCI (MD = 0.253, *p* < .001) and AD (MD = 0.225, *p* = .001), as compared to NC (Table 6). Similarly, pairwise comparisons between the regions revealed significant decrease in PVC–GSH levels in both ACC (MD = 1.36, *p* < .001) and PCC (MD = 1.28, *p* < .001) region as compared to CINGULATE region (Table 7).

**Table 2:** Results of the GLM showing the effect of group, region and their interaction on the absolute GSH concentration including age and gender as covariates

Source	*df*	Mean square	*F*	*p*‐value
Corrected model	34	4.948***	27.910	<.001
Intercept	1	353.197***	1992.186	<.001
Gender	2	0.655*	3.693	.027
Age	24	0.591***	3.335	<.001
Group	2	2.879***	16.236	<.001
Region	2	59.337***	334.685	<.001
Group × region	4	0.722**	4.075	.004
Error	135	0.177		
Total	170			
Corrected total	169			

*Note*. *R* ^2^= 0.875 (adjusted *R* ^2^ = 0.844).

Abbreviations: ACC, anterior cingulate cortex; AD, Alzheimer's disease; AUC, area under curve; GLM, generalized linear model; GSH, glutathione; MCI, mild cognitive impairment; NC, normal control; PCC, posterior cingulate cortex; PVC, partial volume correction; ROC, receiver operator characteristic.

All significant values were set at *p* < .05 (*p < .05, **p < .01, ***p < .001).

**Table 3:** Results of the GLM showing the effect of group, region and their interaction on the PVC–GSH concentration including age and gender as covariates

Source	*df*	Mean square	*F*	*p*‐value
Corrected model	33	2.66***	25.04	<.001
Intercept	1	309.19***	2,907.05	<.001
Gender	1	0.43*	4.07	.045
Age	24	0.37***	3.51	<.001
Group	2	1.26***	11.85	<.001
Region	2	36.00***	338.47	<.001
Group × region	4	0.10	0.96	.431
Error	155	0.11		
Total	189			
Corrected total	188			

*Note*. *R* ^2^ = 0.842 (adjusted *R* ^2^ = 0.808).

Abbreviations: ACC, anterior cingulate cortex; AD, Alzheimer's disease; AUC, area under curve; GLM, generalized linear model; GSH, glutathione; MCI, mild cognitive impairment; NC, normal control; PCC, posterior cingulate cortex; PVC, partial volume correction; ROC, receiver operator characteristic.

All significant values were set at *p* < .05 (*p < .05, **p < .01, ***p < .001).

**Table 4:** Post hoc comparison for the effect of group on absolute GSH concentration level

Pairwise comparisons					
Group (I)	Group (J)	Mean difference (I‐J)	*SE*	*p*‐value a	95% CI for mean difference^a^
NC	MCI	0.684***	0.126	<.001	[0.379 to 0.989]
	AD	0.534***	0.123	<.001	[0.235 to 0.834]
MCI	AD	−0.149	0.121	.661	[−0.444 to 0.145]
	NC	−0.684***	0.126	<.001	[−0.989 to −0.379]
AD	MCI	0.149	0.121	.661	[−0.145 to 0.444]
	NC	−0.534***	0.123	<.001	[−0.834 to −0.235]

*Note*. Based on estimated marginal means.

a Adjustment for multiple comparisons: Bonferroni correction.

Abbreviations: ACC, anterior cingulate cortex; AD, Alzheimer's disease; AUC, area under curve; GLM, generalized linear model; GSH, glutathione; MCI, mild cognitive impairment; NC, normal control; PCC, posterior cingulate cortex; PVC, partial volume correction; ROC, receiver operator characteristic.

The mean difference significance level is set at *p* < .05 (*p < .05, **p < .01, ***p < .001).

**Table 5:** Post hoc comparison for the effect of region on absolute GSH concentration level

Pairwise comparisons					
Region (I)	Region (J)	Mean difference (I‐J)	*SE*	*p*‐value^a^	95% CI for mean difference^a^
ACC	PCC	−0.271**	0.081	.003	[−0.467 to −0.076]
	CINGULATE	−1.977***	0.082	< .001	[−2.175 to −1.778]
PCC	ACC	0.271**	0.081	.003	[0.076 to 0.467]
	CINGULATE	−1.706***	0.083	< .001	[−1.907 to −1.504]
CINGULATE	ACC	1.977***	0.082	< .001	[1.778 to 2.175]
	PCC	1.706***	0.083	< .001	[1.504 to 1.9107]

*Note*. Based on estimated marginal means.

Abbreviations: ACC, anterior cingulate cortex; AD, Alzheimer's disease; AUC, area under curve; GLM, generalized linear model; GSH, glutathione; MCI, mild cognitive impairment; NC, normal control; PCC, posterior cingulate cortex; PVC, partial volume correction; ROC, receiver operator characteristic.

a Adjustment for multiple comparisons: Bonferroni correction.

The mean difference significance level is set at *p* < 0.05 (*p < .05, **p < .01, ***p < .001).

**Table 6:** Post hoc comparison for the effect of group on PVC–GSH concentration level

Pairwise comparisons					
Group (I)	Group (J)	Mean difference (I‐J)	*SE*	*p*‐value^a^	95% CI for mean difference^a^
NC	MCI	0.253***	0.066	<.001	[0.123 to 0.382]
	AD	0.225**	0.066	.001	[0.094 to 0.356]
MCI	AD	−0.028	0.072	.697	[−0.170 to 0.114]
	NC	−0.253***	0.066	<.001	[−0.382 to −0.123]
AD	MCI	0.028	0.072	.697	[−0.114 to 0.170]
	NC	−0.225**	0.066	.001	[−0.356 to −0.094]

*Note*. Based on estimated marginal means.

Abbreviations: ACC, anterior cingulate cortex; AD, Alzheimer's disease; AUC, area under curve; GSH, glutathione; MCI, mild cognitive impairment; NC, normal control; PCC, posterior cingulate cortex; PVC, partial volume correction; ROC, receiver operator characteristic.

a Adjustment for multiple comparisons: Bonferroni correction.

The mean difference significance level is set at *p* < .05 (*p < .05, **p < .01, ***p < .001).

**Table 7:** Post hoc comparison for the effect of region on PVC–GSH concentration level

Pairwise comparisons					
Region (I)	Region (J)	Mean difference (I‐J)	*SE*	*p*‐value^a^	95% CI for mean difference^a^
ACC	PCC	−0.8**	0.059	.582	[−0.22 to −0.07]
	CINGULATE	−1.36***	0.059	<.001	[−1.50 to −1.22]
PCC	ACC	0.08	0.059	.582	[−0.07 to 0.22]
	CINGULATE	−1.2***	0.059	<.001	[−1.43 to −1.14]
CINGULATE	ACC	1.36***	0.059	<.001	[1.23 to 1.50]
	PCC	1.28***	0.059	<.001	[1.14 to 1.91]

*Note*. Based on estimated marginal means.

Abbreviations: ACC, anterior cingulate cortex; AD, Alzheimer's disease; AUC, area under curve; GSH, glutathione; MCI, mild cognitive impairment; NC, normal control; PCC, posterior cingulate cortex; PVC, partial volume correction; ROC, receiver operator characteristic.

a Adjustment for multiple comparisons: Bonferroni correction.

The mean difference significance level is set at *p* < .05 (*p < .05, **p < .01, ***p < .001).

### 3.5 ROC analysis: Diagnostic accuracy of NC, MCI, and AD groups using cingulate GSH level

**Figure 6:**
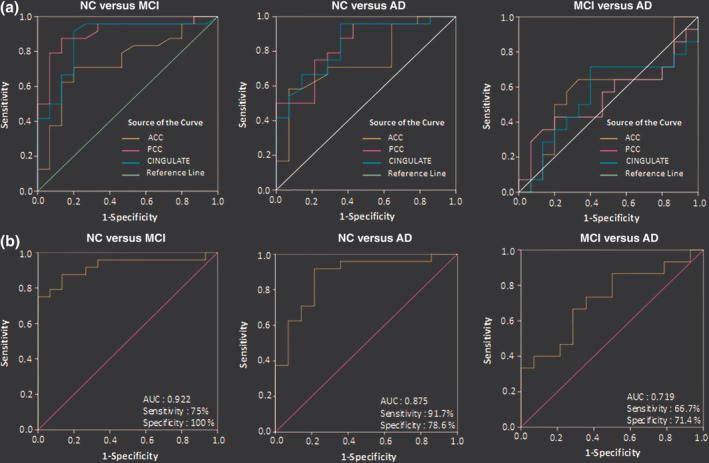
Diagnostic accuracy test using ROC curves for the independent ACC, PCC, and CINGULATE (includes ACC and PCC regions from the same participant) in age‐matched control (NC), MCI, and AD as independent study groups using (a) GSH‐closed form (GSH_cl_) concentration level as single variable in ACC, PCC, and CINGULATE regions, and (b) multivariate ROC analysis on GSH_cl_ concentration levels to indicate combined hypometabolism effect of ACC and PCC regions on the disease diagnosis of MCI and AD from the NC, and MCI from AD. Abbreviations: ACC, anterior cingulate cortex; AD, Alzheimer's disease; GSH, glutathione; MCI, mild cognitive impairment; NC, normal control; PCC, posterior cingulate cortex containing; ROC, receiver operator characteristic

**Figure 7:**
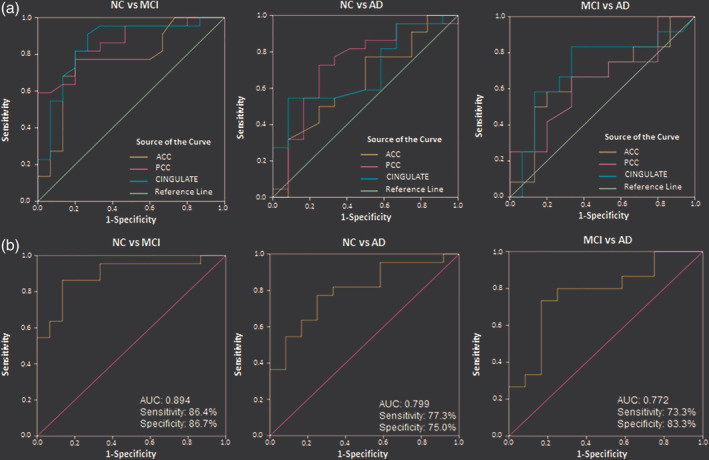
Diagnostic accuracy test using ROC curves for the independent ACC, PCC, and CINGULATE (includes ACC and PCC regions from the same participant) in age‐matched control (NC), MCI, and AD as independent study groups using (a) PVC–GSH_cl_ concentration level as single variable in ACC, PCC, and CINGULATE regions, and (b) multivariate ROC analysis on PVC–GSH_cl_ concentration levels to indicate combined hypometabolism effect of ACC and PCC regions on the disease diagnosis of MCI and AD from the NC, and MCI from AD. Abbreviations: ACC, anterior cingulate cortex; AD, Alzheimer's disease; GSH, glutathione; MCI, mild cognitive impairment; NC, normal control; PCC, posterior cingulate cortex; ROC, receiver operator characteristic

ROC curve analysis was performed to assess the diagnostic accuracy of absolute GSH (Figure 6a, and Table 8) as well as PVC–GSH (Figure 7a, and Table 9) concentration levels in the ACC, PCC, and CC regions for differentiating MCI and AD from NC. Absolute GSH levels from ACC region were differentiating MCI from NC group with 76.2% accuracy and AD from NC group with 79.7% accuracy, whereas GSH levels in PCC region showed the improved classification of MCI group with an accuracy 87.0% from the NC group (Table 8). Similar analysis on the tissue corrected GSH levels revealed ACC region differentiating MCI from NC group with an accuracy 71.2% and AD from NC group with 66.1% accuracy. Meanwhile, GSH levels in PCC region showed the improved classification of MCI group with an accuracy of 78.3% as well as AD group with an accuracy of 77.5% from the NC group (Table 9). However, in both the above analysis, none of the regions differentiated MCI from AD group. Higher accuracy for classification of AD than MCI as compared to NC was observed with GSH level depletion in ACC region, while PCC and CC regions classify MCI better than AD from NC participants indicating the early involvement of PCC region in AD pathology.

**Table 8:** Diagnostic test for absolute GSH concentration in the ACC, PCC, and cingulate regions independently to differentiate NC versus MCI, NC versus AD, and MCI versus AD groups using ROC curve analysis

Univariate ROC characteristics	NC versus MCI	NC versus AD	MCI versus AD
ACC			
No. participants (*N*) (negative/positive groups)	44 (26/18)	42 (26/16)	34 (18/16)
AUC [95% CI]	0.742* [0.581–0.903]	0.747* [0.586–0.908]	0.574 [0.352–0.796]
*p*‐value	.012	.012	.499
Youden index	1.705	1.889	1.595
Sensitivity [95% CI]	70.8% [57.4–84.2]	58.3% [43.3–73.1]	64.3% [48.2–80.4]
Specificity [95% CI]	80.0% [68.2–91.8]	92.9% [85.1–100.0]	66.7% [50.9–82.5]
Accuracy (%)	76.2	79.7	65.6
PCC			
No. participants (*N*) (negative/positive groups)	41 (25/16)	41 (25/16)	32 (16/16)
AUC [95% CI]	0.907*** [0.809–1.000]	0.833** [0.702–0.965]	0.540 [0.319–0.762]
*p*‐value	<.001	.001	.711
Youden index	2.064	2.169	1.964
Sensitivity [95% CI]	87.5% [77.4–97.6]	75.0% [61.7–88.3]	42.9% [25.8–60.0]
Specificity [95% CI]	86.7% [76.3–97.1]	78.6% [66.0–91.1]	80.0% [66.1–93.9]
Accuracy (%)	87.0	77.2	60.9
CINGULATE			
No. participants (*N*) (negative/positive groups)	39 (24/15)	38 (24/14)	29 (15/14)
AUC [95% CI]	0.872*** [0.751–0.994]	0.841** [0.712–0.970]	0.552 [0.331–0.774]
*p*‐value	<.001	.001	.631
Youden index	3.664	3.585	3.259
Sensitivity [95% CI]	91.7% [83.0–100.0]	95.8% [89.4–100.0]	71.4% [54.9–87.8]
Specificity [95% CI]	80.0% [67.4–92.6]	64.3% [49.0–79.5]	60.0% [42.2–77.8]
Accuracy (%)	84.5	75.9	65.5

Abbreviations: ACC, anterior cingulate cortex; AD, Alzheimer's disease; AUC, area under curve; GSH, glutathione; MCI, mild cognitive impairment; NC, normal control; PCC, posterior cingulate cortex containing ; PVC, partial volume correction; ROC, receiver operator characteristic.

All significant values were set at *p* < .05 (*p < .05, **p < .01, ***p < .001).

**Table 9:** Diagnostic test for PVC–GSH concentration in the ACC, PCC, and cingulate regions independently to differentiate NC versus MCI, NC versus AD, and MCI versus AD groups using ROC curve analysis

Univariate ROC characteristics	NC versus MCI	NC versus AD	MCI versus AD
ACC			
No. participants (*N*) (negative/positive groups)	43 (25/18)	42 (25/17)	35 (18/17)
AUC [95% CI]	0.765** [0.602–0.928]	0.633 [0.432–0.833]	0.656 [0.438–0.873]
*p*‐value	.007	.207	.111
Youden index	1.29	1.31	1.24
Sensitivity [95% CI]	75.0% [53.3–90.2]	75.0% [53.3–90.2]	55.6% [30.8–78.5]
Specificity [95% CI]	66.7% [41.0–86.7]	57.1% [28.9–82.3]	60.0% [32.3–83.7]
Accuracy (%)	71.2	66.1	57.8
PCC			
No. participants (*N*) (negative/positive groups)	40 (24/16)	38 (24/14)	30 (16/14)
AUC [95% CI]	0.865*** [0.750–0.980]	0.733* [0.545–0.921]	0.644 [0.429–0.860]
*p*‐value	<.001	.027	.205
Youden index	1.60	1.51	1.60
Sensitivity [95% CI]	56.5% [34.5–76.8]	69.6% [47.1–86.8]	100.0% [79.4–100.0]
Specificity [95% CI]	100.0% [41.0–86.7]	84.6% [54.6–98.1]	21.5% [4.7–50.8]
Accuracy (%)	78.3	77.5	61.0
CINGULATE			
No. participants (*N*) (negative/positive groups)	38 (23/15)	38 (23/15)	29 (15/14)
AUC [95% CI]	0.858*** [0.726–0.989]	0.693 [0.511–0.875]	0.714 [0.504–0.924]
*p*‐value	<.001	.093	.107
Youden index	2.67	3.09	2.47
Sensitivity [95% CI]	90.9% [70.8–98.9]	54.6% [32.2–75.6]	66.7% [38.4–88.2]
Specificity [95% CI]	73.3% [44.9–92.2]	91.7% [61.5–99.8]	88.3% [51.6–97.9]
Accuracy (%)	82.8	73.5	77.5

Abbreviations: ACC, anterior cingulate cortex; AD, Alzheimer's disease; AUC, area under curve; GSH, glutathione; MCI, mild cognitive impairment; NC, normal control; PCC, posterior cingulate cortex; PVC, partial volume correction; ROC, receiver operator characteristic.

### 3.6 Multivariate ROC analysis for the combined effect of GSH alteration in ACC and PCC regions

ROC curve analysis was used for summarizing discriminatory power of a binary prediction model for the combined effect of two regions, that is, ACC and PCC, with age and gender as two covariates. Predicted probabilities were created using binary logistic regression for each combination of group variables, that is, NC versus MCI, NC versus AD, and MCI versus AD using analysis with absolute GSH (Figure 6b, and Table 10) as well as the PVC–GSH concentration levels (Figure 7b, and Table 11). Relaxation time corrected absolute GSH concentration levels as obtained from calculated probability values in multivariate ROC analysis differentiated MCI (*p* < .001) and AD (*p* < .001) groups from NC with improved accuracy of 90.3% and 83.4%, respectively, while the classification accuracy of MCI from AD (*p* = .045) was obtained as 69.1% (Table 10). The PVC–GSH levels in multivariate ROC analysis differentiated MCI (*p* < .001) and AD (*p* = .004) groups from NC with an accuracy of 86.6% and 76.4%, respectively, while the classification accuracy of MCI from AD (*p* = .017) was obtained as 78.5% (Table 11).

**Table 10:** Diagnostic test for the combined effect of absolute GSH concentrations in the ACC and PCC regions to differentiate NC versus MCI, NC versus AD, and MCI versus AD groups using multivariate ROC curve analysis

Multivariate ROC characteristics	NC versus MCI	NC versus AD	MCI versus AD
No. participants (*N*) (negative/positive groups)	39 (24/15)	38 (24/14)	28 (14/14)
AUC [95% CI]	0.922*** [0.834–1.000]	0.875*** [0.756–0.994]	0.719* [0.531–0.907]
*p*‐value	<.001	<.001	.045
Youden index	0.787	0.560	0.586
Sensitivity [95% CI]	75% [61.4–88.6]	91.7% [82.9–100.0]	66.7% [49.2–84.2]
Specificity [95% CI]	100% [−]	78.6% [65.6–91.6]	71.4% [54.7–88.1]
Accuracy (%)	90.3	83.4	69.1

Abbreviations: ACC, anterior cingulate cortex; AD, Alzheimer's disease; AUC, area under curve; GSH, glutathione; MCI, mild cognitive impairment; NC, normal control; PCC, posterior cingulate cortex; ROC, receiver operator characteristic.

All significant values were set at *p* < .05 (*p < .05, **p < .01, ***p < .001).

**Table 11:** Diagnostic test for the combined effect of PVC–GSH concentration levels in the ACC and PCC regions to differentiate NC versus MCI, NC versus AD, and MCI versus AD groups using multivariate ROC curve analysis

Multivariate ROC characteristics	NC versus MCI	NC versus AD	MCI versus AD
No. participants (*N*) (negative/positive groups)	38 (23/15)	38 (23/15)	29 (15/14)
AUC [95% CI]	0.894*** [0.788–1.000]	0.799** [0.648–0.951]	0.772* [0.588–0.956]
*p*‐value	<.001	.004	.017
Youden index	0.55	0.61	0.59
Sensitivity [95% CI]	86.4% [65.1–97.1]	77.3% [54.6–92.2]	73.3% [44.9–92.2]
Specificity [95% CI]	86.7% [59.5–98.3]	75.0% [42.8–94.5]	83.3% [51.6–97.9]
Accuracy (%)	86.6	76.4	78.5

Abbreviations: ACC, anterior cingulate cortex; AD, Alzheimer's disease; AUC, area under curve; GSH, glutathione; MCI, mild cognitive impairment; NC, normal control; PCC, posterior cingulate cortex; PVC, partial volume correction; ROC, receiver operator characteristic.

All significant values were set at *p* < .05 (*p < .05, **p < .01, ***p < .001).

## 4 DISCUSSION

### 4.1 Significance of cingulate region in cognitive functioning

Roles of the HP (during encoding) and the medial temporal lobe (during recall) have been studied as the two prime regions for new memory formation (Chai, Ofen, Gabrieli, & Whitfield‐Gabrieli, 2014; Prince, Daselaar, & Cabeza, 2005). Early AD pathology symptom of recent or short‐term memory decline has also been associated with damage of HP. However, with the observation that older memories could still be consciously recollected led the concept of information storage in a distributed cortical network. To further understand this, researchers have investigated the dynamic control of DMN activity and its importance in cognitive functioning in AD pathogenesis (Leech & Sharp, 2014; Yu et al., 2017). The PCC region has been reported as a highly anatomically connected area of the brain (Hagmann et al., 2008) and the central part of the DMN activities (Buckner, Andrews‐Hanna, & Schacter, 2008; Leech, Kamourieh, Beckmann, & Sharp, 2011).

Increased activity over PCC has been observed when individuals retrieve autobiographical memories or planning for the future, as well as under unconstrained rest when the brain activity is only due to the cognitively free‐wheeling thoughts (Mason et al., 2007). Apart from a role in planning and retrieval, a different study provided evidence that PCC plays a more direct role in regulating the focus of attention (Hahn, Ross, & Stein, 2007). Studies have supported that the magnitude of changes in PCC activity is related to cognitive load in the healthy brain (Singh & Fawcett, 2008) and failure has been associated with inefficient cognitive function (Leech et al., 2011). Disorientation for time and place has been correlated with memory impairment in probable AD patients having minimal to moderate severity of disease (Hirono et al., 1998). The ACC region is functionally coordinated and strongly connected to PCC regions with prominent WM tract (Noonan, Mars, Sallet, Dunbar, & Fellows, 2018). Another study demonstrated the involvement of the ACC region primarily with executive functions in attention, while the PCC having a role in the orientation and interpretation of surroundings (Sutherland, Whishaw, & Kolb, 1988). A study on old memory recall process in rodents also showed that the ACC is necessary for recalling behaviors learned in the past, but not for the same behaviors learned recently, whereas the disruption of ACC was found to selectively impair remote recall (Weible, 2013).

Noninvasive imaging studies also investigated the role of cingulate regions in executive control, emotion, pain, and episodic memory, and have reported associative abnormalities in memory decline in the MCI and AD pathogenesis (Bubb, Metzler‐Baddeley, & Aggleton, 2018). fMRI studies on the cognitive role of CC have shown increased activity in ACC during tasks involving cognitive controls, such as attention, executive functioning and working memory (Lenartowicz & McIntosh, 2005), while the damage of PCC was found to cause trouble with solving cognitive control tasks (Ries et al., 2006). Episodic memory performance related to structural changes within DMN regions also supported the reduced deactivation in task‐based fMRI and its connectivity decline using resting‐state fMRI (rsfMRI) (Bayram, Caldwell, & Banks, 2018). Directed functional connectivity of brain DMN regions and its alteration using rsfMRI was also reported for its role in AD early diagnosis (Yu et al., 2017). This study conducted on both AD and MCI groups showed reduced connectivity from the whole brain to the PCC for regions outside the DMN as compared with the NC group (Yu et al., 2017). Also, AD with APOE ε4 carriers have supported the role of cingulate regions in verbal memory decline associative to the reduced anterior and posterior connectivity as in whole brain functional dynamics (Goveas et al., 2013).

A morphometric study on 29 right‐handed healthy females (mean age 67.7 ± 5.1 years) demonstrated significant correlations between the relative size of the ACC and PCC regions with overall performance and the amount of different types of errors in neuropsychological memory tests (Kozlovskiy, Alexandar, Maria, Nikonova, & Velichkovsky, 2013). Another study including healthy, age‐matched men and women, aged 20–87 years (*N* = 70) examined gender‐specific patterns of age‐related volume decline for cingulate GM and showed significant gray volume loss with age as well as gender differences in structure and function (Mann et al., 2011). Episodic memory decline has also been associated with both the GM atrophy and thinning of the PCC region (Dore et al., 2013; Irish, Addis, Hodges, & Piguet, 2012). While AD is a disease primarily associated with GM loss, the WM changes in different brain regions have also been reported with a role in cognitive expression (Dore et al., 2013). Supportive studies using diffusion tensor imaging metrics to characterize brain WM reported an association between fiber density and memory (Gyebnar et al., 2018; Remy, Vayssiere, Saint‐Aubert, Barbeau, & Pariente, 2015) with reduced brain volume in PCC region and with worsened memory in aMCI (Meyer et al., 2013). These morphometric studies of CC region for its involvement in cognitive processes have also been investigated and found supportive of other functional, directional, and psychological studies in a clinical setting.

### 4.2 Hypo‐functionality of the cingulate region with progressive AD pathology

Reduced metabolism associated with the spatial distribution of fibrillar amyloid deposition and brain atrophy in the nodes of the DMN has been suggested as an early feature of AD (Buckner et al., 2005). Prominent amyloid deposition in the PCC region and its subsequent spread throughout the neocortex, including ACC and the temporal lobe as a spatial distribution within the DMN was reported to be a neuropathological cascade leading to AD (Buckner et al., 2009; Liang et al., 2008; Pardo, Lee, & Alzheimer's Disease Neuroimaging, 2018). Furthermore, a transcriptomics study on autopsy brains of early stage AD revealed significantly lower expression of 70% of the nuclear genes encoding subunits of the mitochondrial electron transport chain in the PCC region. This emphasizes the higher susceptibility of PCC region for hypometabolism in comparison to other key brain regions (Liang et al., 2008). This was further associated with the cerebral metabolic rate for glucose abnormalities using fluorodeoxyglucose–positron emission tomography (FDG–PET) study of AD (Liang et al., 2008). Studies have also stated that in human, cerebral blood flow and metabolic rate are 40% greater than average within the PCC (Raichle et al., 2001). Disruption of this high rate of metabolism in the PCC has been correlated with cognitive state and its response to disorientation for time and place in patients with AD (Hirono et al., 1998; Raichle et al., 2001).

WM tract damage in the connections of the DMN has been correlated with age‐related changes in PCC (Andrews‐Hanna et al., 2007). Clinically, normal older individuals (mean age 73.1 ± 7.8 years) with high levels of amyloid deposition in the PCC showed a negative correlation with functional connectivity (Hedden et al., 2009). Hypoperfusion in the PCC has been reported consistently in all stages of AD (i.e., pre‐MCI, MCI, and fully developed AD) (Sierra‐Marcos, 2017). The certainty of regional functionality using arterial spin labeling studies on MCI and AD also depict similar regions of perfusion changes, particularly hypoperfusion in the PCC region (Sierra‐Marcos, 2017). Investigation on functional connectivity within the DMN found a reduction in early AD, affecting particularly the connection between the PCC and the HP (Greicius, Srivastava, Reiss, & Menon, 2004). Such altered patterns of the PCC functional connectivity were also reported with APOE gene status using the topology of WM connectivity (Raj, Kuceyeski, & Weiner, 2012).

Multimodal imaging studies reported glucose hypometabolism in AD to be associated with the decreased GM atrophy in the ACC, but not in PCC (Sabbagh et al., 2015). Another study involving 47 participants (17 AD, 17 aMCI, and 13 HCs) using combined PET and MRI, reported disrupted glucose metabolism and atrophy in the CC using FreeSurfer (software suite for processing and analyzing human brain MRI images) (Bailly et al., 2015). Lower PCC (*p* < .05) volume was observed with significantly smaller ^18^F‐FDG uptake in the ACC (*p* < .05) as well as PCC (*p* < .001) regions among MCI and AD groups than HC. No significant difference was observed for hypometabolism in ACC but found in PCC (*p* < .05) between AD and MCI patients (Bailly et al., 2015). Studies with integrative neuropsychological, anatomical, and metabolic information have further validated the structural and functional abnormality in the PCC region (Leech & Sharp, 2014; Mutlu et al., 2016). An MRS study also showed a positive correlation between GSH depletion in the HP regions and the executive brain function using trail making (B‐A) test scores among MCI and AD patients (Mandal et al., 2015).

Glucose metabolism and functional connectivity were studied emphasizing the role of CC hypometabolism in AD pathogenesis. However, a gap of studies has been found on antioxidant metabolism in cingulate regions in the AD pathology. Till date, no study has reported the hypometabolism of antioxidants on the MCI and AD cohorts. Role of antioxidants like GSH is critical in combating the OS and biometal homeostasis in AD pathogenesis, and this emphasizes the investigation of GSH alteration, particularly in the CC regions among cognitively impaired groups.

### 4.3 GSH monitoring using plasma, autopsy, and in vivo studies in NC, MCI, and AD groups

A clinical study reported a significant decrease in plasma GSH levels in MCI (*N* = 34) and AD (*N* = 45) compared to age‐matched controls (*N* = 28) (Bermejo et al., 2008). GSH depletion in age and age‐associated disorders has also been supported by different brain autopsy studies (Liu, Wang, Shenvi, Hagen, & Liu, 2004; Venkateshappa et al., 2012). An autopsy study involving AD, PD, and DLB reported that the GSH level in the cingulate region of AD patients is reduced by 49% as compared to age‐matched controls while this specific change was not found in PD and DLB patients (Gu et al., 1998); however, a decreased GSH level with age in HP (*N* = 25) and FC (*N* = 31) has also been reported (Venkateshappa et al., 2012). Furthermore, GSH concentrations in the normal healthy brain (*N* = 5) were reported to be 1.18 ± 0.09 and 0.89 ± 0.03 mM, respectively, from the WM and the GM of parietal cortex regions (Slivka, Spina, & Cohen, 1987). Another postmortem study in MCI, mild and, severe AD brains revealed depleted GSH levels in postmitochondrial supernatant, mitochondrial, and synaptosomal fractions from the FC region, as compared to controls (Ansari & Scheff, 2010). Autopsy as well as neuroimaging studies have reported about the close association of the GSH distribution in the human brain with gender, age, disease condition, and anatomical area. GSH depletion in the HP and the FC regions were correlated (*p* < .001) with the decline in cognitive functions (Mandal et al., 2015). The ROC analysis showed that hippocampal GSH depletion distinguishes between MCI and elderly HCs with 87.5% sensitivity, 100% specificity, whereas in the cortical region, GSH level differentiates MCI and AD with 91.7% sensitivity, 100% specificity. Significant GSH reduction, selectively in the FC and HP brain regions was found to be affected by AD pathology, but not in the cerebellum among AD patients (Mandal et al., 2015).

Evidence presented so far emphasizes the critical role of ACC and PCC regions in cognitive functioning and their affinity to AD pathology. However, to the best of our knowledge, the association of the ACC and PCC regions has not yet been explored for the region‐specific alteration of GSH with its protective role in OS load. In this study, we presented the antioxidant hypometabolism with the quantified GSH levels in both ACC and PCC regions using MEGA‐PRESS sequence, where PCC shows higher depletion of GSH than the ACC region in both MCI and AD patients, as compared to NC.

## 5 POTENTIAL OF CINGULATE METABOLIC ALTERATIONS AS AN EARLY PREDICTIVE BIOMARKER FOR NC TO MCI CONVERSION

^1^H‐MRS has been used extensively to investigate the regional relationships and metabolic alterations in normal aging and AD pathogenesis. GSH monitoring with special editing MEGA‐PRESS sequence in the HP and FC regions provided critical information on the underlying molecular processes of AD pathology. GSH depletion in HP has been suggested as a diagnostic biomarker to differentiate HC from MCI, and similarly the FC region for MCI and AD differentiation (Mandal, 2012; Mandal et al., 2015). The GSH_cl_ change in the HP and FC regions for different age groups (20–40; 41–60, and 61 years and above) has also been reported; however, no significant result in HP was observed among the three study groups (Shukla, Tripathi, & Mandal, 2018). It may be noted that depletion of GSH in HP is very selective and specific to AD pathology as no change in cerebellum GSH levels were found as compared to age‐matched controls (Mandal et al., 2015). In another MRS study involving MCI subjects, it was found that GSH level in both the ACC and PCC regions increases in MCI patients compared to HCs and this increase in GSH level was explained as an early compensatory or neuroprotective response (Duffy et al., 2014). However, in the same study, the GSH peak was identified with PRESS pulse sequence using a 3 T scanner, where, GSH peak appeared in close proximity of high amplitude peak. The probable anomaly of increased GSH level in MCI is likely due to the use of a PRESS sequence for detection of the β‐CH_2_ signal of GSH, which is partly overlapped by intense signals arising from Cr, thus complicating the accurate measurement of GSH. On contrary, our present study shows a significant depletion of the GSH_cl_ level in the ACC (*p* = .023) and PCC (*p* < .001) regions of MCI and ACC (*p* = .008) and PCC (*p* < .001) regions of AD as compared to NC. However, the tissue corrected GSH levels resulted in significant change only in PCC (*p* = .001) region but not in ACC (*p* = .194) among MCI group as compared to NC. Whereas in AD group as compared to NC, the tissue corrected GSH levels significantly differ in both ACC (*p* = .025) and PCC (*p* = .028) regions. Our results also support the literature on autopsy and hypometabolism studies discussed earlier (Bailly et al., 2015) (Gu et al., 1998). Results presented in this study provide further evidence of GSH depletion in AD pathology and that remains selective to the brain regions. The MEGA‐PRESS pulse sequence, on the other hand, is a very selective and confirmatory method to detect GSH peak. Hence, in any in vivo GSH quantitation study, MEGA‐PRESS sequence could be the method of choice of MRS (Mandal, Shukla, Tripathi, & Ersland, 2019; Mischley et al., 2016; Tumati et al., 2018).

Apart from the in vivo GSH, other ^1^H‐MRS metabolites from ACC and PCC regions using PRESS sequence have also been studied in the literature. Lower NAA/Cr and higher myo‐inositol (mI)/Cr in the ACC region have been reported in depressive AD patients comparative to nondepressed AD patients (Guo et al., 2015). Another ^1^H MRS study on absolute metabolite levels reported reduced NAA (*p* < .01) in the ACC but not in PCC and also a significant change in the Cr (*p* = .01) in the PCC region of aMCI. Changes in these neurometabolites with healthy aging have also been reported with significant positive correlation of Cho (*r* = 0.545, *p* = .002), Cr (*r* = 0.571, *p* = .001), and NAA (*r* = 0.674, *p* < .001) in the ACC; and Cho (*r* = 0.614, *p* < .001), Cr (*r* = 0.670, *p* < .001), and NAA (*r* = 0.528, *p* = .003) in the PCC; and NAA (*r* = 0.409, *p* = .025) in the LH, with age (Chiu et al., 2014). Recently, a ^1^H‐MRS study on 7 T using PRESS sequence among HC and MCI patients reported significant changes of GABA (*p* < .005), mI (*p* < .001) in ACC and GABA (*p* < .009), Glu (*p* < .024), and mI (*p* < .010) in PCC regions and no significant change in GSH was observed in either of these regions (Oeltzschner et al., 2019). On the contrary, the reduced glutamate (Glu), glutamine (Gln), and GSH, and an increase in Cr levels of PCC region have been reported as age‐related changes (Suri et al., 2017). Such changes in the Cr level (which was mostly considered to remain unaltered) in AD pathology implies that the use of internal metabolite as a quantitation reference should be avoided (Suri et al., 2017). External referencing using phantom calibration curve is the most suitable choice for absolute metabolic quantitation. A systematic review on neurochemical changes in the aging brain also reported that NAA concentration was consistently reduced with age predominantly in the frontal lobe, whereas the mI concentration increased with age consistently in the PCC (Cleeland, Pipingas, Scholey, & White, 2019). Besides all the above‐mentioned MRS studies, the NMR analysis of a tissue sample from the FC of AD brain also showed a significant decrease in NAA and Cho levels (Zhang et al., 2018).

In this work, we have shown significant depletion of Cho (*p* < .001), Cr (*p* < .001), and NAA (*p* < .001) in both the ACC and PCC regions for MCI as compared to NC (Figure 4). Our results for Cho level changes agree with the NMR study on FC tissue sample (Zhang et al., 2018) and the depletion in both NAA and Cr levels could be due to the damaged brain cells in diseased populations. These results further emphasize the association of metabolic changes in CC with the mild cognitive decline as an early sign of AD. Depletion in the Cho, Cr, and NAA levels were significant in NC to MCI disease transition. The PCC region was found to be more susceptible than the ACC region toward the changes in Cr and NAA levels with early disease progression.

## 6 LIMITATIONS

Published literature on the relative Cho and Cr levels in AD pathology showed disagreement in findings within different MRS‐based (using PRESS) as well as tissue‐based in vitro studies. Also, inconsistencies in GSH levels using PRESS sequence were found with either increased (Duffy et al., 2014), reduced (Suri et al., 2017), or no change (Oeltzschner et al., 2019) in AD pathology. The variability in findings may be due to the presence of GSH peak within the close proximity of strong Cr peak. In order to overcome the ambiguity on metabolic alterations in different brain regions among healthy with different age groups and varying pathological conditions, further studies with consistent MRS/NMR experimental protocol is desired.

Furthermore, in our previous multicenter study, we showed that the two conformer forms of GSH (GSH_cl_ and GSH_ex_) can be simultaneously detected with optimized and specific parameter selection in MEGA‐PRESS sequence (i.e., ON = 4.40/4.56 ppm, OFF = 5.0 ppm, and TE = 130 ms) (Shukla, Mandal, et al., 2018; Shukla, Tripathi, & Mandal, 2018). In this study with parameter specific to GSH_cl_ (ON = 4.40 ppm, OFF = 5.0 ppm, and TE = 120 ms), the GSH_ex_ peak was indistinct and appeared out of phase with GSH_cl_ peak (Shukla, Mandal, et al., 2018; Shukla, Tripathi, & Mandal, 2018). The quantitation of the GSH_ex_ in the present study (Figure S1) found to be unaltered over the NC, MCI, and AD study groups. Further investigation on the relative changes of both conformer forms of GSH among normal aging and different pathological conditions with the specific experimental protocol (TE = 130 ms) is in progress.

## 7 CONCLUSION

This study provides conclusive evidence that GSH depletes significantly in CC regions of DMN, thus emphasizing the role of OS in AD pathology. In PCC region, the magnitude of GSH reduction among MCI patients when compared to NC was found to be higher, than in the ACC region. The GSH change in CC region was found to be similar with our earlier observation in the HP and FC regions, which also supports the hypometabolism of the antioxidant mechanism. To the best of our knowledge, no study is available which investigates the GSH alteration in both ACC and PCC regions of MCI and AD patients using MEGA‐PRESS. Also, no study till date has demonstrated the concurrent changes in GSH and NAA, Cr, Cho metabolites in both ACC and PCC regions using MEGA‐PRESS sequence. The present research has opened up a new area to monitor the alteration of GSH_cl_ and GSH_ex_ forms in healthy and different pathological conditions, which is active ongoing research at NINS laboratory, NBRC, India. Further longitudinal investigation with larger sample size is urgently needed to detect the causal changes in the two different GSH conformers with AD progression. Moreover, a clinical trial is already planned at NINS laboratory and proposed to assess the impact of oral GSH supplementation among MCI patients to improve their cognitive performance.

## CONFLICT OF INTEREST

All the authors declare that there is no personal, academic, financial, or any other conflicts of interest associated with this study.

## Supporting information


**Figure S1** (A) Detection and quantitation of in vivo GSH‐closed form (GSH_cl_) and GSH‐extended form (GSH_ex_) from the fitted peak of respective phase corrected edited‐difference spectra of MEGA‐PRESS from PCC region of age‐matched control (NC). (B) Changes in estimated GSH_ex_ peak areas in the NC, MCI and AD participants in the ACC, PCC and CINGULATE regions.

**Figure S2** Comparison of ^1^H‐MRS peaks for Cho, Cr and NAA detected using PRESS sequence and averaged‐OFF spectra of MEGA‐PRESS from the PCC region of healthy young control.

**Figure S3** Comparison of GSH‐closed form (GSH_cl_) peak areas (mentioned in Figure 3) in (A) ACC and (B) PCC region from age‐matched control (NC), MCI and AD participants.

**Figure S4** External calibration as fitted linear curve for in vitro GSH quantitation using GSH peak values obtained from MEGA‐PRESS sequence on the phantom containing different GSH molar concentrations (1–5 mM).

## Data Availability

The data that support the findings of this study are available on request from the corresponding author. The data are not publicly available due to privacy or ethical restrictions.
