# Supplementary figures and images for: Quantitation of in vivo brain glutathione conformers in cingulate cortex among age‐matched control, MCI, and AD patients using MEGA‐PRESS
Source: Hum Brain Mapp. 2019 Oct 4;41(1):194–217. doi: 10.1002/hbm.24799 (PMC7268069; doi:10.1002/hbm.24799)

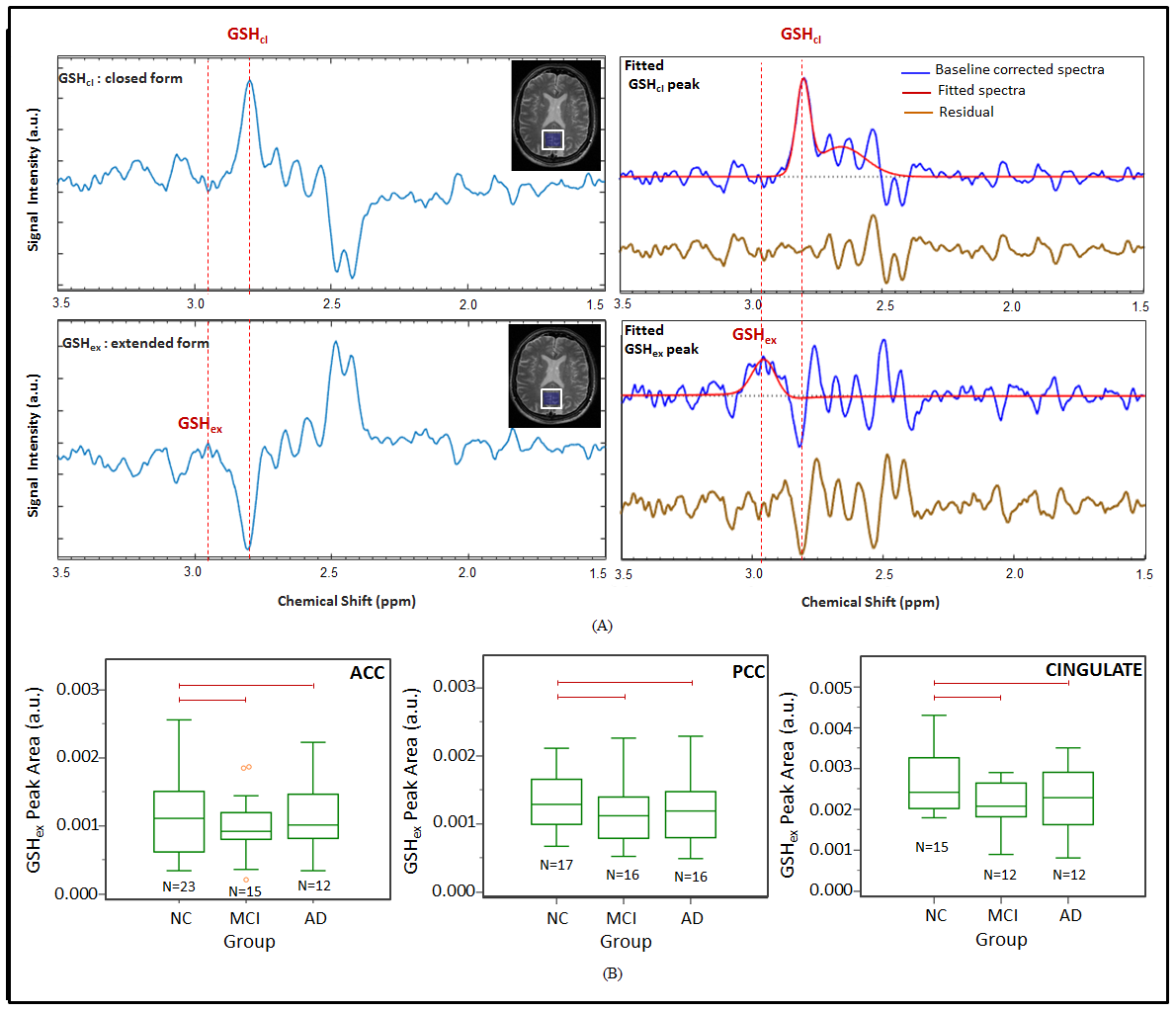

Supplement: Supplementary file 1 — Figure S1 (A) Detection and quantitation of in vivo GSH‐closed form (GSHcl) and GSH‐extended form (GSHex) from the fitted peak of respective phase corrected edited‐difference spectra of MEGA‐PRESS from PCC region of age‐matched control (NC). (B) Changes in estimated GSHex peak areas in the NC, MCI and AD participants in the ACC, PCC and CINGULATE regions. [file HBM-41-194-s001.tif]

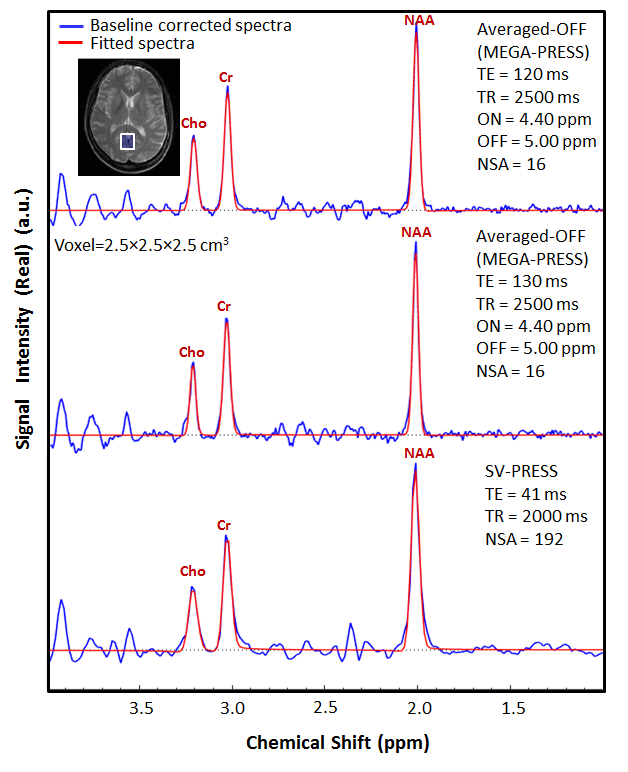

Supplement: Supplementary file 2 — Figure S2 Comparison of 1H‐MRS peaks for Cho, Cr and NAA detected using PRESS sequence and averaged‐OFF spectra of MEGA‐PRESS from the PCC region of healthy young control. [file HBM-41-194-s002.tif]

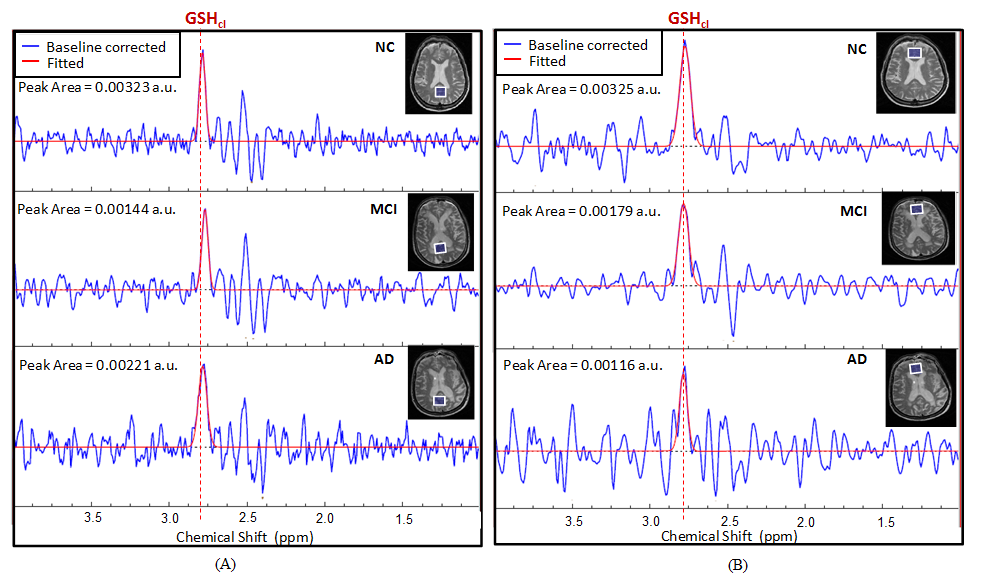

Supplement: Supplementary file 3 — Figure S3 Comparison of GSH‐closed form (GSHcl) peak areas (mentioned in Figure 3) in (A) ACC and (B) PCC region from age‐matched control (NC), MCI and AD participants. [file HBM-41-194-s003.tif]

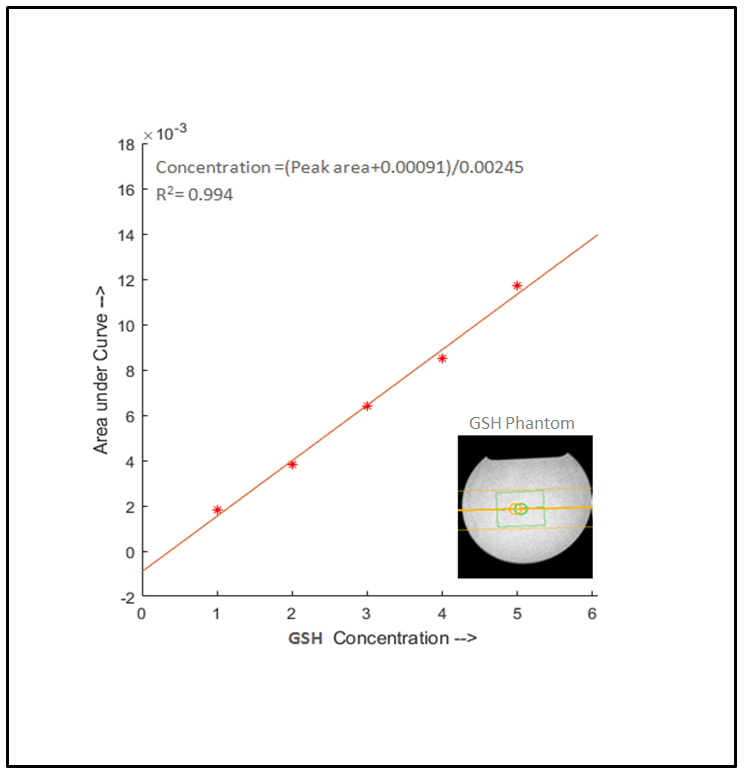

Supplement: Supplementary file 4 — Figure S4 External calibration as fitted linear curve for in vitro GSH quantitation using GSH peak values obtained from MEGA‐PRESS sequence on the phantom containing different GSH molar concentrations (1–5 mM). [file HBM-41-194-s004.tif]
